# The Impact of Anxiety and Depression on the Quality of Life of Hemodialysis Patients

**DOI:** 10.5539/gjhs.v8n1p45

**Published:** 2015-05-15

**Authors:** Chrysoula Vasilopoulou, Eirini Bourtsi, Sophia Giaple, Ioannis Koutelekos, Paraskevi Theofilou, Maria Polikandrioti

**Affiliations:** 1 Department of Nursing, TEI of Athens, Greece; 2 Health Psychology, Panteion University, Department of Psychology, Athens, Greece

**Keywords:** anxiety, depression, quality of life, hemodialysis patients

## Abstract

**Material and Methods:**

The sample studied consisted of 395 hemodialysis patients. Data was collected by the completion of a specially designed questionnaire for the needs of the present study which apart from socio-demographic and clinical, it also included HAD_S_ scale to assess the level of anxiety and depression as well as the scale Missoula-VITAS Quality of Life Index (MVQOLI) to assess patients’ quality of life.

**Results:**

The results of this study showed that 47.8% had high anxiety levels and 38.2% had high levels of depression. The average total score of quality of life was found to be 17.14. It was also shown that the total score of quality of life presented statistically significant association with family status (p=0.007), educational level (p<0.001), the number of children (p=0.001), patients’ adherence to doctors’ orders (p=0.003) and proposed diet (p=0.002) and the relations of patients with healthcare professionals and the other patients (p<0.001). The multiple linear regression showed that the overall quality of life score was statistically associated with the levels of depression after adjusted for possible confounders. More specifically, it was found that total score of quality of life was 2.5 and 4.4 points lower for patients with moderate and high levels of depression, respectively, compared to patients with low levels of depression (p<0.001).

**Conclusions:**

Evaluation of anxiety and depression in conjunction with quality of life in hemodialysis patients should be an integral part of the therapeutic regimen.

## 1. Introduction

It is widely accepted that end stage renal disease patients experience various problems due to medical illness as well as psychological problems that exert a negative influence on the outcome of the disease. Anxiety and depression are the most common psychiatric disorders that goes in parallel with renal failure (Turkistani, Nuqali, Badawi, Taibah, Alserihy, Morad, & Kalantan, 2014; Feroze, Martin, Reina-Patton, Kalantar-Zadeh, & Kopple, 2010; Stein, Cox, Afifi, Belik, & Sareen, 2006).

Levin was first to introduce the term “psychonephrology,” so as to highlight that patients undergoing renal replacement therapy usually encounter with multiple stressors thus resulting in psychiatric disturbance (Levy, 2008).

According to the literature, a variety of factors seem to trigger or worsen an already established anxiety and depression. The main factors are physical and cognitive impairment, restrictions in daily life, compliance to therapeutic regimen including restrictions in diet, fatigue, the fear of death, failure to fulfill prior roles in family, society or in work as well as dependency upon treatment and health professionals (Chilcot, Wellsted, Da Silva-Gane, & Farrington, 2008; Cukor, Coplan, Brown, Friedman, Cromwell-Smith, Peterson, & Kimmel, 2007; Theofilou, 2013; McCann & Boore, 2000; Watnick, Wang, Demadura, & Ganzini, 2005).

Interestingly, health professionals focus on managing the biological dimension of the disease and usually underestimate symptoms from mental dimension. This effort becomes highly confounded since symptoms of anxiety and depression usually overlap with the clinical symptomology of kidney disease, mainly uremic state. For instance, components of depression such as anorexia, fatigue, sexual and sleep disturbances share common characteristics with uremic state. Consequently, anxiety and depression extend the physical and cognitive impairment that experience hemodialysis patients and contribute significantly to the increase of hospitalization rate and use of health care services. According to estimates, 20-30% of dialysis patients experience depression, thus making an imperative need, the evaluation of depression in clinical routine (Chilcot et al., 2008; Cukor et al., 2007; Theofilou, 2013; McCann & Boore, 2000; Watnick et al., 2005; Cohen, Norris, Acquaviva, Peterson, & Kimmel, 2007). Anxiety and depression affect negatively the quality of hemodialysis patients’ life (Unruh & Hess, 2007; Avramovic & Stefanovic, 2012).

The aim of the present study was to explore the impact of anxiety and depression on the quality of life of hemodialysis patients.

## 2. Material & Methods

### 2.1 Study Design

The sample studied consisted of 395 patients (222 men and 173 women) undergoing hemodialysis. This sample was a convenience sample.

The study included all patients who met the inclusion criteria during the study period June 2014 to December 2014 and participated in the study after they had been orally informed and given consent. Criteria for enrolling a patient in the study were: comprehension of Greek language and being under hemodialysis. 

The data collected for each patient included: a) socio-demographic characteristics: gender, age, marital status, education level, place of residence and number of children, b) clinical characteristics: if the patient was suffering from any other illness, the level of awareness of the health status, the years undergoing hemodialysis, the frequency and duration of hemodialysis, as well as information on how strictly they comply with treatment guidelines and the proposed diet and c) other variables such as the relation with the physicians and nurses, the relations with the social and family environment, whether they concealed the problem from the community, if they reported themselves as anxious and if they had help at home.

### 2.2 Mental Health Assessment

For the evaluation of the mental health of the patients, the scale that was used was “The Hospital Anxiety And Depression Scale (HADS)”. This scale was proposed in 1983 by Zigmond AS & Snaith RP (Zigmond & Snaith, 1983). The HADS scale consists of 14 questions, of which seven evaluate the level of depression (questions 2, 4, 6, 8, 10, 12 and 14) and the other seven evaluate the anxiety level (questions 1, 3, 5, 7, 9, 11 and 13) of the respondents. The range of the total score of anxiety and depression level is between 0 and 21. In addition, for both scores it has been proposed and the widely used in the literature following classification: 0-7 indicating no anxiety or depression, respectively, score 8-10 indicating moderate levels of anxiety or depression, respectively, and score> 11 indicates high levels of anxiety / depression. The HADs had high reliability and validity in Greek population by Mystakidou et al. in cancer patients (Mstakidou, Tsilika, Parpa, Katsouda, Galanos, & lahos, 2004) and by Michopoulos et al. (2008) in general hospital sample.

### 2.3 Quality of Life Assessment

To evaluate the quality of life of the patients the scale Missoula-VITAS Quality of Life Index (MVQOLI) was used. This scale has been translated and it was culturally adapted to the Greek data by Mrs. Theofilou et al. (Theofilou, Kapsalis, & Panagiotaki, 2012). Although there are two versions of the scale, one with 25 and a second one with 15 questions, in this survey the one with the 15 questions was used. This scale assesses five dimensions of quality of patients’ life, the symptoms, functioning, interpersonal relationships, well-being and transcendent. For each dimension, three types of information are collected: (a) assessment (subjective measurement of the actual situation), (b) evaluation (degree of acceptance of the real situation) and (c) importance (the extent to which this dimension affects the actual quality of life).

The questions of each dimension expressing the “assessment” were graded in a 5-degree Likert scale from -2 to 2. Questions expressing “evaluation” were graded from -4 to 4, and the questions that express the “importance” were graded from 1 to 5. To calculate the total score of each dimension of quality of life, the scores of “appreciation” and “evaluation” were added and then multiplied this sum by the degree of “importance” ((estimate + evaluation) x importance). The total score of each dimension reflects the extent that this dimension affects the quality of life of patients. Higher scores indicate better quality of life. The average score of total quality of life ranged from 0 to 30.

### 2.4 Statistical Analysis

Normality tests of continuous variables were performed, using the Kolmogorov-Smirnov test and histograms. Nominal variables are presented using frequencies and percentages, whereas the continuous variables are presented with means and standard deviation or medians and interquartile range.

One-way ANOVA or Kruskal-Wallis test was used to test the existence of correlation between a quantitative continuous variable following the normal distribution or not, respectively, and a nominal variable with more than two categories. Also, independent samples t-test and the Mann-Whitney test was used to check the existence of correlation between a quantitative continuous variable following the normal distribution or not, respectively, and a nominal variable with 2 categories.

Multivariate linear regression was performed to explore the impact of anxiety and depression on patients quality of life after controlling for potential confounders such as socio-demographic factors, data on the underlying disease and the current state of health of participants. The results are presented with beta coefficients and 95% confidence interval (95% CI).

As statistically significant was the observed significance level of 5%. All statistical analyzes were performed with version 20 of SPSS program (SPSS Inc, Chicago, Il, USA).

## 3. Results

### 3.1 Patients’ Characteristics

Table 1 presents the socio-demographic and clinical characteristics of the participants.

**Table 1:** Patients’ Characteristics

Characteristics		Frequency	Percent
**Sex (male)**		222	56.2
**Age**	**30-40**	48	12.2
	**41-50**	71	18.0
	**51-60**	69	17.5
	**61-70**	91	23.0
	**71-80**	97	24.6
	**<30**	19	4.8

**Family status**	**Married**	198	50.1
	**Single**	83	21.0
	**Divorced**	18	4.6
	**Widowed**	86	21.8
	**living together**	10	2.5

**Education level**	**Elemetary**	153	38.7
	**Secondary**	123	31.1
	**University**	103	26.1
	**Msc-Phd**	16	4.1

**Job**	**Unemployed**	33	8.4
	**Civil servant**	46	11.6
	**Private employee**	51	12.9
	**Freelancer**	39	9.9
	**Domestic**	67	17.0
	**Pensioner**	156	39.5
	**Other**	3	.8

**Residence**	**Attica**	100	25.3
	**County capital**	179	45.3
	**Small city**	48	12.2
	**Countryside**	68	17.2

**No of Children**	**None**	109	27.6
	**One**	116	29.4
	**Two**	130	32.9
	**>two**	40	10.1

**Co-morbidities (yes)**		173	43.8

**Years from first hemodialysis**	**<=1**	41	10.4
	**2-5**	143	36.2
	**6-10**	138	34.9
	**11-15**	60	15.2
	**>16**	13	3.3

**Frequency of hemodialysis (per week)**	**2**	7	1.8
	**3**	378	95.7
	**4**	10	2.5

**Informed of the state of their health**	**Very much**	118	29.9
	**Enough**	233	59.0
	**A little**	42	10.6
	**Not at all**	2	0.5

**Compliance with doctor’s advices**	**Very much**	109	27.6
	**Enough**	154	39.0
	**A little**	124	31.4
	**Not at all**	8	2.0

**Compliance with the proposed diet**	**Very much**	99	25.1
	**Enough**	137	34.7
	**A little**	131	33.2
	**Not at all**	28	7.1

**Relation with nursing staff**	**Very good**	241	61.0
	**Good**	126	31.9
	**Moderate**	28	7.1

**Relation with medical staff**	**Very good**	222	56.2
	**Good**	123	31.1
	**Moderate**	49	12.4
	**Bad**	1	0.3

**Relation with other patients**	**Very good**	151	38.2
	**Good**	137	34.7
	**Moderate**	84	21.3
	**Bad**	21	5.3
	**Very bad**	2	.5

**Change in body image (yes)**		269	68.1

**Body image influence the behaviour of others towards you (yes)**		158	40,0

**Consider yourself anxious (Yes)**		220	55.7

**Difficulties in relations with social environment**	**Very much**	3	.8
	**Enough**	34	8.6
	**A little**	226	57.2
	**Not at all**	132	33.4

**Difficulties in relations with family environment**	**Very much**	13	3.3
	**Enough**	45	11.4
	**A little**	125	31.6
	**Not at all**	212	53.7

**Hiding the problem from social environment (Yes)**		132	33,4

**Other person at home. who helps in everyday tasks (Yes)**		300	75,9

### 3.2 Anxiety and Depression

**Figure 1:**
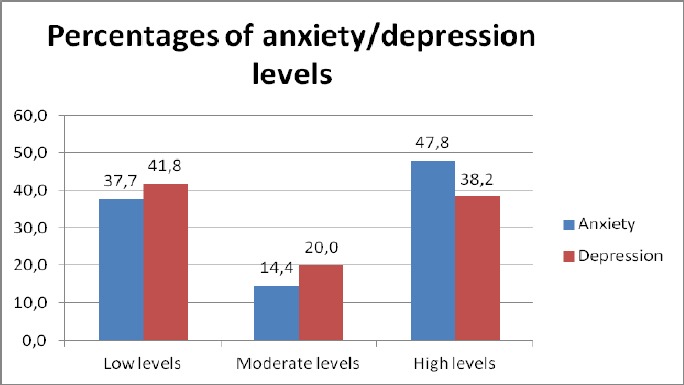
Anxiety and depression levels

Figure 1 illustrates that the majority of participants experienced high levels of anxiety (47.8%), while in terms of depression, the majority was found to experience low levels of depression (41.8%) although high was the percentage of patients suffering from high level of depression (38.2%), too.

### 3.3 Quality of Life

The average total score of quality of life was found to be 17.14. Means or medians of all five dimensions are presented in Table 2 also.

**Table 2:** Quality of life scores

	Score Mean±SD
**Symptom**	3.30(11.15)
**Function**	8(-3,16)*
**Interpersonal**	16(0,20)*
**Well-Being**	-8(-12,9)*
**Trascendent**	2.48(15.21)
**Total score**	**17.14(4.83)**

* data are presented using median (25^th^, 75^th^ percentile).

Table 3 shows the association of the total score of quality of life with various characteristics. It is observed that the total score of quality of life presents statistically significant association with family status, educational level and number of children. Specifically, the average total score of quality of life was higher in married people (p=0.007). Also, the average total score of quality of life of patients studied in university was higher than the others (p < 0.001). Moreover, a lower quality of life scores were found in patients that do not have children (p=0.001). Additionally, there is a statistically significant correlation between the total score of quality of life and patient information about the problem of health (p < 0.001). The mean total score of quality of life of patients who were not aware of the problem of health, was less than the patients who were very informed. A statistically significant correlation was found between the total score of quality of life and how strictly the patients followed the doctors’ orders and proposed diet (p=0.003 & p=0.002, respectively). Specifically, the quality of life of patients who did not follow at all or they followed a little the instructions and the proposed diet was lower than in other patients. Statistically significant association was shown between the total score of quality of life and the relation of patients with medical/nursing staff and the other patients (p < 0.001). More specifically, the average total score of quality of life for patients who had very good relation with the medical and nursing staff and other patients was greater than in other patients. Moreover, there was a statistically significant correlation between the total score of quality of life and social issues such as the difficulties in the relations with social and family environment, hiding the problem from their social environment and the existence of home assistance for everyday activities. In more details, the average quality of life was greater for patients who did not have any difficulties in their family or social environment (p < 0.001), for those who did not hide their health problems from the community (p < 0.001) and for those who had home help for handling everyday (p < 0.001).

**Table 3:** Factors associated with quality of life

Characteristics		Mean± SD	p-value
**Sex**	**Male**	17.3± 4.69	0.458
	**Female**	16.93± 5.01	

**Age**	**<40**	17.39± 5.46	0.114
	**41-60**	17.7± 4.85	
	**61-80**	16.6± 4.53	

**Family status**	**Married**	17.86± 4.6	**0.007**
	**Single**	16.01± 5.09*	
	**Other**	16.71± 4.86	

**Education level**	**Elementary**	16.21± 4.53	**<0.001**
	**Secondary**	16.91± 4.62	
	**University**	18.56± 5.12*§	

**Job**	**Employee**	17.95± 5.24	0.137
	**Pensioner**	17.01± 4.37	
	**Other**	16.71± 4.98	

**Residence**	**Attica**	16.65± 4.57	0.102
	**County capital**	17.71± 5.38	
	**Small city/Countryside**	16.68± 4.03	

**No of Children**	**None**	15.72± 4.97	**0.001**
	**One**	18.13± 5.14*	
	**>=two**	17.36± 4.32*	

**Years from first hemodialysis**	**<=5**	17.39± 5.09	0.106
	**6-10**	17.37± 4.35	
	**>=11**	16.05± 4.94	

**Frequency of hemodialysis (per week)**	**2**	13.09± 4.3	0.050
	**3**	17.26± 4.83	
	**4**	15.43± 3.91	

**Co-morbidities**	**Yes**	16.68± 4.51	0.098
	**No**	17.49± 5.05	

**Informed of the state of their health**	**Very much**	18.34± 5.17	**<0.001**
	**Enough**	17.02± 4.55*	
	**A little/Not at all**	14.49± 4.28^*§^	

**Compliance with doctor’s advice**	**Very much**	17.98± 4.76	**0.003**
	**Enough**	17.52± 4.82	
	**A Little/Not at all**	15.99± 4.72^*§^	

**Compliance with the proposed diet**	**Very much**	18.3± 5.13	**0.002**
	**Enough**	17.46± 4.59	
	**A Little**	16.33± 4.53*	
	**Not at all**	15.21± 5.25*	

**Relation with nursing staff**	**Very good**	18.3± 4.7	**<0.001**
	**Good**	15.99± 4.32*	
	**Moderate**	12.27± 3.81^*§^	

**Relation with medical staff**	**Very good**	18.28± 4.64	**<0.001**
	**Good**	16.66± 4.47*	
	**Moderate/Bad**	13.23± 4.32^*§^	

**Relation with other patients**	**Very good**	19.08± 4.69	**<0.001**
	**Good**	17.15± 4.19*	
	**Moderate**	14.59± 4.5^*§^	
	**Bad/very bad**	13.6± 4.41^*§^	

**Change in body image**	**Yes**	16.24± 4.8	**<0.001**
	**No**	19.06± 4.33	

**Body image influence the behaviour of others towards you**	**Yes**	15.37± 4.92	**<0.001**
	**No**	18.32± 4.4	

**Consider yourself anxious**	**Yes**	14.61± 3.77	**<0.001**
	**No**	20.31± 4.09	

**Difficulties in relations with social environment**	**Very/Enough**	12.65± 2.95	**<0.001**
	**A little**	17.18± 4.81*	
	**Not at all**	18.32± 4.58*	

**Difficulties in relations with family environment**	**Very/Enough**	12.6± 2.74	**<0.001**
	**A little**	15.54± 4.39*	
	**Not at all**	19.32± 4.27^*§^	

**Hiding the problem from social environment**	**Yes**	15.73± 4.95	**<0.001**
	**No**	17.84± 4.62	

**Other person at home who helps in everyday tasks**	**Yes**	17.99± 4.63	**<0.001**
	**No**	14.44± 4.47	

* statistically significant difference with 1st category, after Bonferroni correction;

§ statistically significant difference with 2nd category, after Bonferroni correction.

### 3.4 Anxiety, Depression and Quality of Life

Table 4 represents the association between the levels of anxiety or depression and patients’ quality of life. It was found that there was statistically significant association (p<0.001 respectively). More specifically patients with low levels of anxiety or depression had better quality of life compared to those with moderate or high levels of anxiety or depression.

**Table 4:** Association between Anxiety/depression and quality of life

Anxiety/Depression		Mean± SD	p-value
**Anxiety**	Low Anxiety levels	20.75± 3.76	**<0.001**
	Moderate Anxiety levels	18± 3.78*	
	High Anxiety levels	14.02± 3.64*§	

**Depression**	Low depression levels	20.96± 3.28	**<0.001**
	Moderate depression levels	16.69± 3.24*	
	High depression levels	13.18± 3.49*§	

* statistically significant difference with 1st category, after Bonferroni correction;

§ statistically significant difference with 2nd category, after Bonferroni correction.

### 3.5 Multivariate Linear Regression

The multiple linear regression (Table 5) showed that the overall quality of life score is statistically associated with the levels of depression after adjusted for possible confounders. More specifically quality of life is 2.5 and 4.4 points lower for patients with moderate and high levels of depression respectively than patients with low levels of depression (p < 0.001 respectively).

**Table 5:** Multivariate linear regression

		β-coefficient (95% CI)	p-value	Adjusted* β-coefficient (95% CI)	p-value
**Anxiety**	**Low Anxiety levels**	Ref		ref	
	**Moderate Anxiety levels**	-2.753(-3.886,-1.619)	**<0.001**	-0.27(-1.25,0.72)	0.596
	**High Anxiety levels**	-6.726(-7.524,-5.929)	**<0.001**	-0.85(-1.97,0.27)	0.136
**Depression**	**Low depression levels**	Ref		ref	
	**Moderate depression levels**	-4.272(-5.174,-3.371)	**<0.001**	-2.59(-3.51,-1.68)	**<0.001**
	**High depression levels**	-7.779(-8.521,-7.037)	**<0.001**	-4.39(-5.47,-3.32)	**<0.001**

* after adjusting for all the statistically significant factors: Family status, Education level, No of Children, Informed of the state of their health, Compliance with doctor’s advice, Compliance with the proposed diet, Relation with nursing staff, medical staff and other patients, Change in body image, Consider yourself anxious, Difficulties in relations with social and family environment, Hiding the problem from social environment and Having other person at home who helps in everyday tasks.

## 4. Discussion

The results of the present study showed that 47.8% of the participants experienced high level of anxiety while 38.2% experienced high level of depression. The average total score of quality of life was found to be 17.14. Prevalence of anxiety and depression vary in research studies due to different instruments and methodology used, thus not allowing comparisons between different populations, globally (Preljevic, Østhus, Os, Sandvik, Opjordsmoen, Nordhus, & Dammen, 2013; Lee, M. S. Kim, Cho, & S. R. Kim, 2013; Birmelé, Le Gall, Sautenet, Aguerre, & Camus, 2012; Park et al., 2010; Kimmel & Patel, 2006).

Regarding anxiety, depression and quality of life, it was found that patients with low levels of anxiety or depression had better quality of life. The multiple linear regression showed that the overall quality of life score was statistically associated with the levels of depression after adjusted for possible confounders. More specifically quality of life is 2.5 and 4.4 points lower for patients with moderate and high levels of depression respectively when compared to other patients.

According to the literature, mainly depression is common to hemodialysis patients. Furthermore, decreased health related of life and increased levels of depression share common socio-demographic and clinical characteristics (Preljevic et al., 2013; Lee et al., 2013; Birmelé et al., 2012; Park et al., 2010; Kimmel & Patel, 2006).

Park et al. (2010) showed that 31.9% of 160 hemodialysis patients experienced depression. The same researchers also showed inverse linear relation between depression and health related quality. Moreover, it was also shown that clinical and socio-demographic characteristics were associated with depression and health related quality of life. More in detail, advanced age (>60 years old), low hemoglobin level (<10g/dl) and low economic status were associated with depression whereas advanced age, female gender, diabetes mellitus, high comorbidity and hypoalbuminemia were associated with decreased health related quality of life.

Drayer et al. (2006) who interviewed 62 hemodialysis outpatients showed that patients of younger age were depressed and reported lower quality of life. Cruz et al. (Cruz, Fleck, & Polanczyk, 2010) claimed that depression is a predictor index for low quality of life. Olaqunju et al. (Olaqunju, Campbell, & Adeyemi, 2015) who explored the association between anxiety/depression an the quality of life in 100 endstage renal disease patients showed that employment, married status, young age, and cost of treatment were related positively with quality of life. Moreover, anxiety/depression were independently related to quality of life. The results by Olaqunju et al. (2015) are similar with the present study which showed that the overall quality of life score was statistically associated with the levels of depression.

Given that depressive symptoms are associated with impaired quality of life, Finkelstein et al. (F. O. Finkelstein, Wuerth, & S. H. Finkelstein, 2010) and Hmwe et al. (Hmwe, Subramanian, Tan, & Chong, 2015) supported that early recognition and treatment of depression is a matter of high importance in hemodialysis patients.

Feroze et al. (Feroze, Martin, Reina-Patton, Kalantar-Zadeh, & Kopple, 2010) claimed that the psychiatric burden experienced end stage renal disease patients exert a negative effect on both quality of life and treatment.

A possibly responsible factor for the association between high levels of anxiety or depression and poor quality of life is attributed to poor compliance to therapy. More in detail, according to Ossareh et al., (Ossareh, Tabrizian, Zebarjadi, & Joodat, 2014) who explored depression in 150 hemodialysis patients claimed that a possible explanation for high levels of depression in hemodialysis patients was non adherence to medication while adherence or non-adherence to the therapeutic regimen was not significantly related to quality of life. However, treatment with antidepressants improved both quality of life and depression. Akman et al. (Akman, Uyar, Afsar, Sezer, Ozdemir, & Haberal, 2007) suggested that early diagnosis of depression in patients waiting for renal transplant contributes to the improvement of their quality of life.

Iyasere et al. (Iyasere, & Brown, 2014) showed that depression is a non-renal determinant of quality of life in older end stage renal disease patients, thus coming to similar conclusions with the present study which highlight the impact of anxiety and depression in hemodialysis patients.

## 5. Conclusions

Quality of life score was associated with the levels of depression after adjusted for possible confounders. More specifically, quality of life was 2.5 and 4.4 points lower for patients with moderate and high levels of depression,

Measurement of quality of life should incorporate assessment of psychosocial variables in clinical practice and planning of interventional strategies to reduce the burden of illness.

Early intervention in the treatment of depression would have a positive effect on outcome of the disease.

## 6. Limitations of the Study

The study sample was not representative of hemodialysis patients in Greece, but a convenience sample. The relevant sampling method limits the generalizability of results. Also, the fact that the study was cross-sectional is not allowing the emergence of a causal relation between quality of life and socio-demographic and clinical variables.
